# Antibiotic consumption in laboratory confirmed vs. non-confirmed bloodstream infections among very low birth weight neonates in Poland

**DOI:** 10.1186/s12941-017-0196-y

**Published:** 2017-03-31

**Authors:** A. Różańska, J. Wójkowska-Mach, P. Adamski, M. Borszewska-Kornacka, E. Gulczyńska, M. Nowiczewski, E. Helwich, A. Kordek, D. Pawlik, M. Bulanda

**Affiliations:** 1 Chair of Microbiology, Jagiellonian University Medical College, 18 Czysta Street, 31-121 Krakow, Poland; 2 Institute of Nature Conservation Polish Academy of Sciences, Krakow, Poland; 3 Clinic of Neonatology and Intensive Neonatal Care, Warsaw Medical University, Warsaw, Poland; 4 Clinic of Neonatology, Polish Mother’s Memorial Hospital-Research Institute, Lodz, Poland; 5 Clinic of Neonatology and Intensive Neonatal Care, Institute of Mother and Child, Warsaw, Poland; 6 Department of Neonatal Diseases, Pomeranian Medical University, Szczecin, Poland; 7 Clinic of Neonatology, Jagiellonian University Medical College, Krakow, Poland

**Keywords:** Antimicrobial consumption, Infection surveillance, Bloodstream infections, Neonatal infections

## Abstract

**Background:**

Newborns are a population in which antibiotic consumption is extremely high. Targeted antibiotic therapy should help to reduce antibiotics consumption. The aim of this study was an assessment of antibiotic usage in bloodstream infections treatment in the Polish Neonatology Surveillance Network (PNSN) and determining the possibility of applying this kind of data in infection control, especially for the evaluation of standard methods of microbiological diagnostics.

**Methods:**

Data were collected between 01.01.2009 and 31.12.2013 in five teaching NICUs from the PNSN. The duration of treatment in days (DOT) and the defined daily doses (DDD) were used for the assessment of antibiotics consumption.

**Results:**

The median DOT for a single case of BSI amounted to 8.0 days; whereas the median consumption expressed in DDD was 0.130. In the case of laboratory confirmed BSI, median DOT was 8 days, and consumption—0.120 DDD. Median length of therapy was shorter for unconfirmed cases: 7 days, while the consumption of antibiotics was higher—0.140 DDD (p < 0.0001). High consumption of glycopeptides expressed in DOTs was observed in studied population, taking into account etiology of infection.

**Conclusions:**

Even application of classical methods of microbiological diagnostics significantly reduces the consumption of antibiotics expressed by DDD. However, the high consumption of glycopeptides indicates the necessity of applying rapid diagnostic assays. Nevertheless, the assessment of antibiotic consumption in neonatal units represents a methodological challenge and requires the use of different measurement tools.

## Background

Infection control in neonatal intensive care units (NICUs) should have high priority, because its incidence is among the highest in different patient populations. Bloodstream infections (BSI) are the most common clinical form of infections in NICUs. The incidence of early-onset BSI (diagnosed <3 days after delivery) is 7% in Poland [1] and in Norway [2], 6% in the USA [3] and 2.4% in Israel [4].

In contrast, risk of late-onset BSI reaches 14.9/1000 patient days (pds) worldwide [5]; in the German NeoKISS: 8.3/1000 pds [6] and in Poland—6.7/1000 pds [7]. However, in the USA (for infants born at 28 weeks gestation or earlier)—36% [8], while in Israel, 39% [9].

Numerous studies show that newborns are a population in which antibiotic usage is extremely high [10, 11]. Those studies were performed mainly in West European countries and in the United States, but there are no such reports from Poland or Central Europe.

Assessment of antibiotic usage in neonatal intensive care units encounters significant difficulties connected with the lack of standardized methods for this specific patient population. Defined daily dose (DDD), an international standard measure used for drug consumption assessment, is a technical unit of measurement which reflects the average maintenance dose per day for a drug used for its main indication in adults [12]. For this reason, this parameter has certain limitations for the analysis in the child population. However, defined daily dose was used in some studies, especially for comparative purposes in homogenous patient population [13]. Other parameters used for antibiotic usage assessment are as follows: LOT—the number of days during which at least one dose of any antibiotic was received, DOT—the aggregate sum of LOT or PDD—prescribed daily dose or proportion of patients with antibiotic treatment in a specific period [14–16].

Antibiotics consumption assessment can have numerous implications. In the area of infection control it can serve as a relatively simple indicator for assessing the effectiveness of methods of microbiological diagnostics. Microbiological diagnostics of blood stream infections in hospital practice still remains a challenge.

The aims of this study were:

- an assessment of antibiotic usage in bloodstream infection (BSI) treatment, taking into account etiology, in the Polish Neonatal Surveillance Network wards using two kinds of parameters, that is DDD and DOT,
- determining the possibility of applying this kind of data in infection control, especially for the evaluation of standard methods of microbiological diagnostics.

## Methods

Data were collected prospectively between 01.01.2009 and 31.12.2013 in five teaching neonatal intensive care units (NICU) that took part in the Polish Neonatology Surveillance Network (PNSN). The PNSN is a prospective national surveillance system for the most relevant infections in the group of very low birth weight (birth weight <1500 g, VLBW) infants in Poland. The PNSN recorded severe infections, including BSI, observed during hospitalization: from admission to discharge, transfer or death. Participation in PNSN was voluntary and confidential for wards. Detailed description of data collection system, study wards, epidemiology of early- and late-onset BSI and its microbiology have been already published elsewhere [1, 7]. The study was approved by the Bioethics Committee of Jagiellonian University Medical College—no. KBET/221/B/2011. All data entered into the electronic database and analyzed retrospectively during the preparation of this article were previously de-identified. BSI (both: early- and late-onset) were defined according to Gastmeier et al. [17] with modifications. BSI was detected when at least two of the following signs were observed:

Temperature >38 or <36.5 °C or temperature instability, tachycardia or bradycardia, apnea, prolonged capillary refill, metabolic acidosis, hyperglycemia, the other sign of bloodstream infections, such as: lethargy; and one of the following criteria: C-reactive protein (CRP) >2.0 mg/dL, immature/total neutrophil ratio (I/T ratio) >0.2, leukocytes <5000/µL, platelets <10,000/µL.

Early-onset BSI was defined as septicemia diagnosed <3 days after delivery.

Laboratory confirmed BSI (LC-BSI) were those cases in which positive results of the microbiological testing were obtained, that means etiological factor was isolated. All blood specimens of at least 1 mL (taken prior to implementing antibiotic treatment) were injected into an aerobic blood culture bottle. Isolates were identified by the automated identification system (VITEK 2, bioMérieux, Poland). In the studied wards, molecular methods for identification of etiological factors were not used. BSI cases in which samples for microbiological testing were not collected or etiological factor was not isolated were classified as not confirmed ones.

Antibiotic usage for BSI treatment (until cure) was assessed for 767 cases. The analysis of antibiotic use included only the cases in which treatment was successful—13 records concerning infants who had died within 7 days of starting the therapy were excluded from the study (all of them were laboratory confirmed).

Two kinds of indicators were used for the description of antibiotic usage:

1. DOT, expressed in days—the aggregate (for every separate type of antibiotics) sum of number of days during which at least one dose of any antibiotic was received, and
2. DDD, expressed in grams—the defined daily dose, according to the ATC/DDD system of the World Health Organization (Anatomical Therapeutic Chemical, group “J01”) [12].

Both measures were taken into account in reference to one case of infection. Data on the medicine type, dose, and the length of therapy were derived from individual records in the chart of each individual patient.

Antibiotic consumption were calculated for all antimicrobials used in therapy, and for the following classes: beta-lactams (ampicillin, cloxacillin, piperacillin, cefotaxime, ceftriaxone, ceftazidime, meropenem, imipenem), aminoglycosides (amikacin, netilmicin, gentamicin), glycopeptides (vancomycin), antimycotics (fluconazole, amphotericin B) and others (ciprofloxacin, clindamycin, erythromycin, clarithromycin, sulfamethoxazole with trimethoprim). Etiological factors were assigned to the following groups: Gram-negative (*Enterobacteriaceae* and other rods), Gram-positive (staphylococci, streptococci), candida. While conducting treatment with the application of drugs from several groups, used in parallel or consecutively, all of them were included into analysis. When, during treatment, positive microbiological cultures were obtained from different samples, or samples taken at different times (within 5 days), growth of microorganisms belonging to various groups (e.g. *Escherichia coli* and *Candida albicans*) was defined as cases of changing etiology (group “changing”).

Due to DDD and DOT distribution significantly different from the normality, the statistical analysis based on the Kruskal–Wallis test. If the significance had been obtained, analysis was suplemented by the post hoc Steel–Dwass test, with critical value p = 0.05. All analysis were provided with SAS JMP package.

## Results

In the study period, records of 2003 VLBW newborns and 780 BSI cases (regardless of the date of recognition of first BSI symptoms) were filled with all data.

Laboratory confirmed BSI (LC-BSI) constituted 84.9% (662) of all recognized cases of BSI.

In the analyzed population of VLBW neonates with BSI, in whom the etiological agent was isolated, combination therapy was used in 67% of cases, while in the group without microbiological confirmation, in 74% of cases.

The total duration of antibiotic therapy for 767 cases of BSI, which are incorporated into the present analysis, amounted to 14,056 DOTs or 381.6 DDDs. The median length of antibiotic therapy for a single case of BSI, regardless of microbiological confirmation or its lack, amounted to 8.0 days; whereas the median consumption expressed in DDD was 0.130. In the case of LC-BSI, median DOT was also 8.0 days, and consumption—0.120 DDD. Median length of therapy was shorter for unconfirmed cases: 7.0 days, while the consumption of antibiotics was higher—0.140 DDD (p < 0.0001) (Table 1).

**Table 1:** Values (median) of DOT and DDD in bloodstream infections treatment in microbiologically confirmed (LC) vs. unconfirmed cases (Not-LC)

Antimicrobial group	DOT (days)		p value	DDD		p value
	Microbiologically confirmed N = 649	Microbiologically unconfirmed N = 118		Microbiologically confirmed N = 649	Microbiologically unconfirmed N = 118	
	Median (IQR)	Median (IQR)		Median (IQR)	Median (IQR)	
Aminoglycosides	7.0 (5.0; 9.3)	7.0 (6.0;7.0)	0.5265	0.101 (0.050; 0.171)	0.095 (0.045; 0.179)	<0.0001
Antimycotic	12.5 (9.0;16.0)	10.0 (7.8;14.3)		0.210 (0.132; 0.040)	0.198 (0.073; 0.463)	
Beta-lactams	8.0 (5.0;11.0)	7.0 (6.8;8.8)		0.254 (0.136; 0.438)	0.300 (0.210; 0.420)	
Glycopeptides	10.0 (8.0;11.0)	9.0 (7.0;11.0)		0.120 (0.080; 0.168)	0.120 (0.083; 0.146)	
Other	9.0 (6.0;14.8)	8.0 (5.0;15.5)		0.210 (0.105; 0.530)	0.098 (0.060; 0.410)	
Total	8.0 (5.0; 16.0)	7.0 (5.0; 15.5)		0.120 (0.050; 0.530)	0.143 (0.045; 0.463)	

*DOT* days of therapy (per one infection case), *DDD* defined daily dose (per one infection case), *IQR* interquartile range, *N* number of BSI cases

Antibiotic consumption expressed by the DDD index was higher in the case of BSI caused by Gram-positive cocci than Gram-negative bacilli (0.140 vs. 0.136 DDD, Table 2), and the differences concerned 2× higher consumption of aminoglycosides (0.109 vs. 0.056 DDD, Table 2; p = 0.0092, Table 3).

**Table 2:** Values (median) of DOT and DDD in bloodstream infections treatment according to etiological factors

Etiology	DOT (days)	DDD
	Median (IQR)	Median (IQR)
Gram+ N = 452		
Aminoglycosides	7.0 (5.0; 9.0)	0.109 (0.560; 0.180)
Antimycotics	12.0 (9.3; 16.0)	0.195 (0.131; 0.341)
Beta-lactams	7.0 (5.0; 10.0)	0.252 (0.180; 0.420)
Glycopeptides	10.0 (8.0; 11.0)	0.128 (0.088; 0.168)
Other	9.0 (5.0; 15.0)	0.211 (0.096; 0.452)
Total	9.0 (5.0; 15.0)	0.140 (0.056; 0.453)
Gram− N = 103		
Aminoglycosides	6.0 (4.3; 8.0)	0.056 (0.036; 0.137)
Antimycotics	12.0 (8.2; 17.0)	0.228 (0.131; 0.360)
Beta-lactams	8.0 (4.0; 11.0)	0.244 (0.096; 0.448)
Glycopeptides	8.0 (4.0; 9.0)	0.098 (0.039; 0.149)
Other	8.5 (5.3; 10.8)	0.229 (0.099; 0.608)
Total	7.5 (4.0; 17.0)	0.136 (0.036; 0.447)
Changing N = 76		
Aminoglycosides	7.0 (5.0; 10.0)	0.105 (0.061; 0.180)
Antimycotics	12.0 (10.0; 16.0)	0.250 (0.158; 0.735)
Beta-lactams	7.0 (6.5; 11.0)	0.281 (0.169; 0.495)
Glycopeptides	10.0 (7.0; 11.0)	0.085 (0.056; 0.167)
Other	9.0 (6.0; 14.5)	0.187 (0.120; 2.580)
Total	9.0 (5.0; 16.0)	0.149 (0.056; 2.580)
Candida N = 18		
Aminoglycosides	8.5 (4.8; 12.8)	0.140 (0.070; 0.210)
Antimycotics	10.5 (9.0; 15.8)	0.225 (0.092; 0.353)
Beta-lactams	12.0 (9.0; 19.5)	450 (192.9; 2175)
Glycopeptides	10.0 (7.3; 10.8)	0.087 (0.063; 0.161)
Other	13.5 (10.0; 17.0)	3.276 (0.153; 6.400)
Total	10.0 (4.8; 19.5)	0.164 (0.063; 6.400)

Changing—when, during treatment, positive microbiological cultures were obtained from different samples, or samples taken at different times (within 5 days), growth of microorganisms belonging to various groups (e.g. *Escherichia coli* and *Candida albicans*) was defined as cases of changing etiology (group “changing”)

*DOT* days of therapy (per one case), *DDD* defined daily dose (per one case), *IQR* interquartile range, *N* number of cases

**Table 3:**
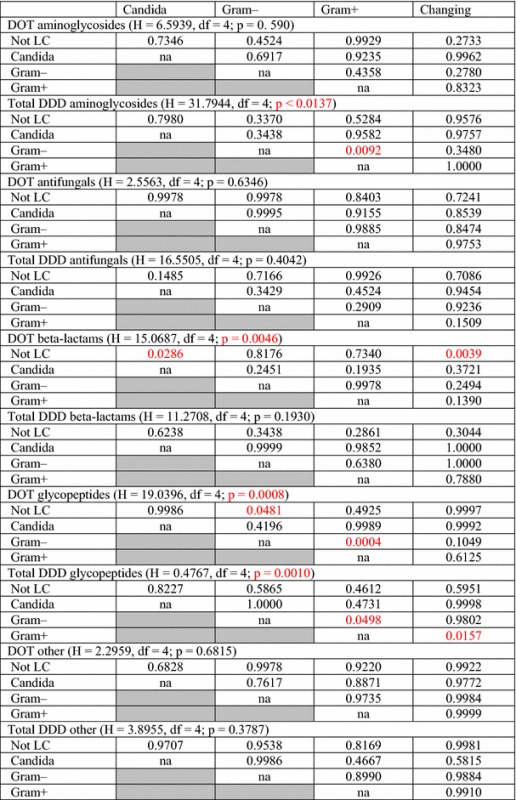
Statistical significance of antibiotic consumption expressed in DOT and DDD in bloodstream infections treatment according to etiological factor

p values for each pair of etiological factor groups are given in the table cells

Changing—when, during treatment, positive microbiological cultures were obtained from different samples, or samples taken at different times (within 5 days), growth of microorganisms belonging to various groups (e.g. *Escherichia coli* and *Candida albicans*) was defined as cases of changing etiology (group “changing”)

*DOT* days of therapy, *DDD* defined daily dose, *IQR* interquartile range, *na* not applicable, *Not LC* not laboratory confirmed

Highest DOT values for beta-lactams concerned fungal infections and for the “changing” group, similarly to the consumption of antibiotics expressed by DDD.

Median length of therapy for BSI infections caused by Gram-positive cocci was longer than the ones caused by Gram-negative bacilli (9.0 vs. 7.5 DOT, Table 2), and the differences were mainly associated with the employment of glycopeptides (8.0 vs. 10.0 DOT, Table 2, p = 0.0004, Table 3).

Detailed data on antibiotic consumption expressed by DDD and DOT (values of medians per one infection case), taking into account groups of antibiotics, are presented in Table 2.

The results of statistical analysis regarding the consumption of the individual groups of antibiotics depending on the etiology of infection are shown in Table 3.

Depending on the applied indicator assessing the consumption of antibiotics in the treatment of BSI: DOT or DDD, the percentage share for individual groups of antibiotics varied.

**Fig. 1:**
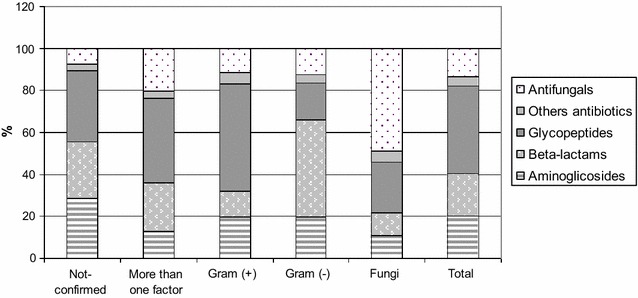
Distribution of antibiotic groups used in the treatment of BSI according to etiology, expressed in DOT

In treatment of BSI as a whole, according to the DOT index, glycopeptides were used the longest: 42.1%, and, after taking into account the etiology of infection, it was the predominant group also in infections caused by Gram-positive cocci: 51%, in the event of changes in the etiological agent: 40.6%. In treatment of microbiologically unconfirmed BSI, glycopeptides were used in 33.8% DOT (Fig. 1).

**Fig. 2:**
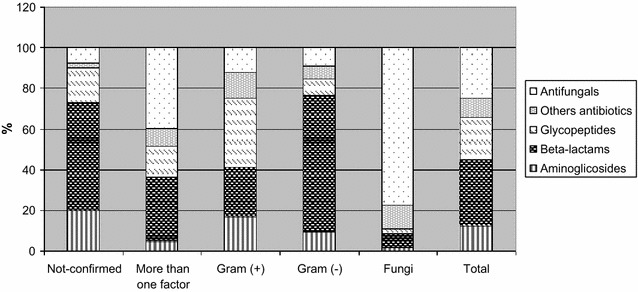
Distribution of antibiotic groups used in the treatment of BSI according to etiology, expressed in DDD

The largest share in total consumption of antibiotics in all analyzed cases of BSI expressed by DDD was represented by beta-lactams: 32.6%, especially with microbiologically unconfirmed BSI: 53.2% (Fig. 2).

In the event when antibiotic consumption was evaluated by DDD, antifungal medication constituted almost one-fourth of the applied drugs, and when the unit of measurement was DOT—13.3% (Figs. 1, 2).

## Discussion

BSI represent a critical complication associated with hospitalization of very low birth weight (VLBW) infants, contributing to longer stay, and different long-term adverse outcomes. This phenomenon is well-understood and described [3, 7, 18–21], contrary to the subject under discussion, in which, unfortunately, data concerning antibiotic consumption are sparse and incomplete.

The proportion of microbiologically confirmed cases of BSI observed in the present study indicates similarity, but not identicalness, with other national programs. In the analysis of differences, attention should be paid to the applied definition of infections and different significance of microbiological findings for various types of surveillance. In American NHSN, in which to confirm LC-BSI, it was necessary to obtain at least 2 identical blood cultures, clinical sepsis was observed in 6.7–12.7% of infections [22], i.e. twice less frequently than in this study—significantly less restrictive in evaluating microbiological results. It unfortunately, indicates too rare application of the capabilities of contemporary microbiology in everyday clinical practice of the studied NICUs. This is confirmed by the results of a Cypriot study, in which LC-BSI constituted 96% of all BSIs [23]. Another matter is the debated problem of legitimacy of repeated blood drawing for cultures from VLBW newborns. Currently, it is more and more frequently assumed that, in this patient population, it is more justifiable to draw a single full-volume sample than to take two or more—even in the event of a result revealing typical skin contaminants, i.e. coagulase-negative staphylococci [24]. This is confirmed by the definitions adopted in the Netherlands [25], in NICHD Vermont Oxford Network [3] and the ones employed in the German national program called Neo-KISS [17].

The obtained data show that the use of diagnostics, even based on the standard, basic level, meaning culture, is a proceeding effectively affecting the consumption of antibiotics, and therefore, the costs of therapy, i.e. they reduce the consumption of antibiotics expressed by DDD.

The indicator most commonly used to assess the consumption of drugs, including antibiotics, is defined daily dose. This recognized international standard applicable in measuring the consumption of antibiotics is based on the average dose for the treatment of adults. For this reason, it is an indicator which, in relation to children, should be used with caution and one should take into account its limitations [26, 27].

Howether, both measures: DDD and the length of therapy were used by different authors with the application of various denominators—the number of admissions, number of person days or with respect to the treatment of a single patient [13, 15].

To evaluate the consumption of antibiotics in pediatric wards, particularly neonatal units, Gerber et al. employed DOT [15]. Depending on the type of neonatal unit, they found the length of treatment to be in the range from 5.7 in medical NICUs to 34.3 DOT in surgical NICUs. Median DOT in this study was about forty percent higher than in the medical NICUs in the study by Gerber; however, due to distinct populations and different degrees of detail, it is difficult to explicitly compare these values. Studies concerning antibiotic consumption in the neonatal population are not numerous, but what is more important: the published papers present a differentiated approach to the subject and are carried out with the use of diverse methodology and for various needs [5, 11, 13, 26, 28].

However, the main objective of this study was the analysis of the evaluation of antibiotic consumption in the treatment of one form of infection in a narrow and specific patient population, giving special consideration to the possibility of its use in the evaluation of the effectiveness and accuracy of microbiological diagnostics as an element of surveillance of infections in NICU.

And so, in NICUs covered by the study, significantly lower consumption of antibiotics expressed by DDD was observed in the case of LC-BSI treatment, compared to those in which the etiological agent was not isolated. On this basis, it can be concluded that the microbiological diagnosis of BSI in newborns treated in NICU was conducted properly and the results of microbiological tests were used in targeted therapy, what made it possible to obtain a reduction in the consumption of antimicrobial drugs and, consequently, the cost of treatment. In Polish NICUs, costs of medication account for nearly one-fifth of the total cost of treatment, the amount of which is inversely proportional to a child’s birth weight and directly proportional to the length of hospitalization [20].

A different situation was observed in the population of the PNSN neonates, who developed necrotising enterocolitis, wherein the length of therapy and consumption rates were not affected by the isolation of the potential etiological agent [29]. But with NEC, difficulties present themselves as regards obtaining material for microbiological examination, which would enable the isolation of the etiological agent. The quoted results concerning antibiotic consumption in NEC cases and the ones demonstrated in the present study regarding BSI illustrate the possibilities of how analyses in this respect could be utilized in infection control and, in particular, in evaluation of adequacy and effectiveness of microbiological diagnostics.

On the other hand, no significant differences in DOT values in cases of LC-BSI and microbiologically unconfirmed BSI were observed. This is contradictory to the current approach to modern antimicrobial stewardship: in newborn population, with suspected BSI, it is recommended to terminate antibiotic treatment after 48 h since the identification of symptoms, if the infection was not confirmed microbiologically. Even traditional diagnostics based on the culture method ensures obtaining a positive result (information on microbial etiology of infection) within 48 h [30, 31].

For antimicrobial stewardship to efficiently and effectively influence the reduction of antibiotic consumption, but not to decrease patient safety, in neonatal units, a principle of daily detailed review of the situation of neonates treated with antibiotics should be introduced, so as to minimize the intake of antibiotics in children, whose blood cultures and other clinical specimens tested negative and symptoms of infection are no longer observed or infectious origin of the disease was excluded. The lack of significant differences in DOT values of laboratory confirmed vs. not-confirmed BSI cases would point to the fact that these recommendations are not applied in the PNSN wards.

It should also be noted that rapid diagnostic molecular methods (which enable rapid assessment of the need to implement or discontinue therapy with vancomycin) could be implemented to decrease glycopeptides consumption, because glycopeptides are not easy to use in neonates [32, 33]. For it has been observed that the use of glycopeptides in the case of BSI caused by *Enterobacteriaceae* was lower by only approx. 20% in comparison with BSI caused by Gram-positive cocci. Generally, in our study, in treatment of BSI as a whole glycopeptides were used the longest. Similar situation was reported in study of Sameer et al. [34].

As for aminoglycosides, only the use of the DDD indicator permitted the demonstration of their significantly increased consumption in BSI caused by Gram-positive cocci (109 DDD), in comparison with BSI that were caused by Gram-negative bacilli (56.1 DDD).

Also noteworthy is the fact that there is a large share of antifungal drugs in the treatment of the analyzed cases of BSI, 25% of the entire consumption expressed by DDD (13.3% DOT), with simultaneous, lower than anticipated, participation of yeast-like fungi isolated in microbiological testing [35, 36]. This coincides with the trends observed in other studies. According to Fridkin, the application of fluconazole in infection prophylaxis contributed to this fact, which is also confirmed by other authors [6]. Thus, the high level of antimycotic medication consumption in PNSN wards fulfilled the task of reducing the incidence of fungal infections.

Antibiotic consumption assessment using at least two different measures, as presented in our study, can be a useful tool in antibiotic stewardship [27]. In presenting case, the results of the analysis indicate the need of implementing more sensitive and faster methods of microbiological diagnostics (PCR and/or MALDI-TOF) as a first step of reducing antibiotics consumption due to faster identification of etiological factor of infection. PCR increases the sensitivity of diagnostics test and shortens the time of identification of microorganisms without culture. MALDI-TOF improves the specificity and shortens the time of identification after receiving microorganism grow in culture method [37, 38]. These diagnostics techniques are still very rarely used in Polish hospital. They are considered as expensive procedures by hospitals’ management, because complex cost-effectiveness analysis in the field of infection control, taking into account at least the cost of prolonged hospital stay, are not performed. In the study patient population occurrence of BSI significantly increases length of stay in NICU, by approximately 20 days [21].

## Conclusions

Analysis of antibiotic consumption is an essential component of infection control, especially for NICU patients—for effective planning and reliable evaluation of interrelationships between individual elements of control programs.

Application of classical methods of microbiological diagnostics based on blood cultures significantly reduces the consumption of antibiotics expressed by DDD.

High consumption of glycopeptides marked by DOT indicates the necessity of applying rapid diagnostic assays.

Nevertheless, the assessment of antibiotic consumption in neonatal units represents a methodological challenge and requires the use of different measurement tools.

## Abbreviations

BSI: bloodstream infection
DOT: days of therapy
DDD: defined daily dose
LC-BSI: laboratory confirmed bloodstream infection
LOT: length of therapy
NICU: neonatal intensive care unit
PDD: prescribed daily dose
PNSN: Polish Neonatal Surveillance Network
VLBW: very low birth weight
PCR: polymerase chain reaction
MALDI-TOF: matrix assisted laser desorption ionisation, MALDI; time of flight, TOF
